# Effect of canakinumab on frailty: A post hoc analysis of the CANTOS trial

**DOI:** 10.1111/acel.14029

**Published:** 2023-11-05

**Authors:** Ariela R. Orkaby, Aerin Thomson, Jean MacFadyen, Richard Besdine, Daniel E. Forman, Thomas G. Travison, Paul M. Ridker

**Affiliations:** ^1^ New England GRECC (Geriatric Research, Education, and Clinical Center) VA Boston Healthcare System Boston Massachusetts USA; ^2^ Division of Aging, Brigham & Women's Hospital Harvard Medical School Boston Massachusetts USA; ^3^ Center for Cardiovascular Disease Prevention and Cardiovascular Division, Brigham & Women's Hospital Harvard Medical School Boston Massachusetts USA; ^4^ Alpert Medical School of Brown University Providence Rhode Island USA; ^5^ Section of Geriatric Cardiology, Department of Medicine (Divisions of Geriatrics and Cardiology) University of Pittsburgh Medical Center Pittsburgh Pennsylvania USA; ^6^ Geriatric Research, Education, and Clinical Center VA Pittsburgh Healthcare System Pittsburgh Pennsylvania USA; ^7^ Marcus Institute for Aging Research, Hebrew SeniorLife Harvard Medical School Boston Massachusetts USA

**Keywords:** frailty, inflammation, trial

## Abstract

Although inflammation is strongly associated with frailty, whether medications that lower inflammation decrease frailty is unclear and randomized trial evidence is scant. We sought to test whether canakinumab, a therapeutic monoclonal antibody that inhibits IL‐1β and reduces C‐reactive protein (CRP), can lower frailty risk. This was a post hoc analysis of the Canakinumab ANti‐inflammatory Thrombosis Outcome Study (CANTOS), a randomized double‐blind placebo‐controlled trial of 10,061 stable postmyocardial infarction patients randomized to subcutaneous canakinumab once every 3 months. Incident frailty was measured using a 34‐item cumulative‐deficit Frailty Index (FI). Time‐to‐event analysis using intent to treat. A total of 9942 CANTOS participants had data to calculate a baseline FI. Median age was 61 (IQR 54–68); 74% were male, 12% Asian, 3% Black, 80% White, and 16% Hispanic/Latino. At baseline, mean FI score was 0.12 and 13% were frail using a cutoff of 0.2. Over 5 years, 1080 participants (12.5%) became frail and mean FI scores increased to 0.14. There was no effect on frailty incidence according to randomization to any canakinumab dose versus placebo over time, HR 1.03 (0.91–1.17), *p* = 0.63. Results were similar using phenotypic frailty. Additionally, the primary findings of CANTOS in terms of canakinumab‐associated cardiovascular event reduction were unchanged in analyses stratified by baseline frailty. In conclusion, among stable adult patients with atherosclerosis, random allocation to interleukin‐1b inhibition with canakinumab versus placebo did not lower risk of incident frailty over 5 years. More randomized data are needed to understand the role of targeted anti‐inflammatory medications for frailty prevention in older adults.

## Graphical abstract

In this post hoc analysis of the CANTOS trial, among stable adult patients with atherosclerosis, interleukin‐1b inhibition with canakinumab versus placebo did not lower the risk of incident frailty over 5 years. More randomized data are needed to understand the role of targeted anti‐inflammatory medications for frailty prevention in older adults.

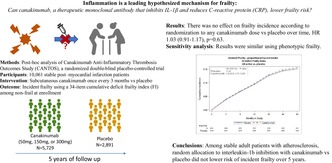

**Abbreviations**
BMI: body mass index
CANTOS: Canakinumab ANti‐inflammatory Thrombosis Outcome Study
CRP: C‐reactive protein
FI: frailty index
HR: hazard ratio
IL‐6: interleukin 6
MACE: major adverse cardiovascular events
MI: myocardial infarction
NSAID: nonsteroidal anti‐inflammatory drugs
SOF: Study of Osteoporotic Fractures

## 1 INTRODUCTION

Frailty is a geriatric syndrome of vulnerability, common in older adults, that increases the risk of cardiovascular events, functional decline, morbidity, and mortality (Afilalo et al., 2014; Clegg et al., 2013; Hoogendijk et al., 2019). A key hypothesized underlying mechanism leading to frailty is inflammation, which has also been implicated in other chronic conditions of aging (Ferrucci & Fabbri, 2018; Jenny, 2012; Lopez‐Otin et al., 2013; Walston et al., 2002). Whether medications that lower inflammation can also lower the risk of frailty is an active area of investigation in which randomized placebo controlled data are scant (Ferrucci & Fabbri, 2018).

Canakinumab is a therapeutic monoclonal antibody that targets IL‐1β blockade leading to reductions in both IL‐6 and the hepatic acute phase reactant C‐reactive protein (CRP). In the recent multinational randomized placebo controlled canakinumab anti‐inflammatory thrombosis outcome study (CANTOS), canakinumab was shown to significantly reduce rates of major adverse cardiovascular events (MACE) (Ridker et al., 2017), as well as incident non‐small‐cell lung cancer, inflammatory anemia, gout, and large joint osteoarthritis events (Ridker, MacFadyen, et al., 2017; Schieker et al., 2020; Solomon et al., 2018; Vallurupalli et al., 2020). The ability of canakinumab to meaningfully reduce each of these common conditions is a direct result of the drugs effect on the inflammatory system. Moreover, each of these conditions increases in prevalence at older ages, are mediated in part by inflammation, and are associated with increased frailty risk (Clegg et al., 2013). Specifically, cardiovascular disease is bidirectionally associated with an increased risk of frailty (Afilalo et al., 2014), yet whether anti‐inflammatory medications that lower risk of cardiovascular disease such as canakinumab can also lower frailty risk has not been rigorously tested in humans although animal studies have been promising (Keller et al., 2019).

The geroscience hypothesis suggests that drugs impacting multiple aspects of aging biology, such as the hallmarks of aging (Lopez‐Otin et al., 2013), will improve healthspan and prevent morbidity (Sierra et al., 2021), making canakinumab a candidate drug for the prevention of aging‐related health decline. We sought on a post hoc basis to examine the effect of canakinumab on frailty among participants in CANTOS. Secondarily, we examined the effect of canakinumab versus placebo for the primary major adverse cardiovascular events endpoint, stratified by frailty. We hypothesized that those randomized to canakinumab would have (1) lower incidence of frailty, (2) improved self‐reported functional status, and (3) the same or greater impact in reduction of cardiovascular events.

## 2 METHODS

### 2.1 Trial design

The CANTOS has been described in detail previously (Ridker et al., 2017). Briefly, this was a multinational, randomized, double‐blind, placebo‐controlled trial of 10,061 stable postmyocardial infarction patients with a high sensitivity (hs) CRP level ≥2 mg/L who were allocated to receive canakinumab (50, 100, or 300 mg) or matching placebo via subcutaneous route every 3 months. The trial was conducted between 2011 and 2017 at 1091 clinical sites in 39 countries. Participants with a history of chronic or recurrent infections, prior cancer other than basal cell skin carcinoma, a suspected or known immunocompromised state, or a history of (or at high risk for) tuberculosis or HIV‐related disease, and those using systemic anti‐inflammatory treatments were excluded. All participants provided written informed consent to participate in the trial, which was overseen by an independent data and safety monitoring board. The results of the main trial as well as its associated protocol and statistical analysis plan, which evaluated the effects of canakinumab versus placebo on incident major adverse cardiovascular events, have been published previously (Ridker et al., 2017).

### 2.2 Outcomes

The primary outcome of this study was incident frailty over the duration of the trial. Secondarily, we assessed change in mean frailty score over time and change in self‐reported functional ability using the EQ‐5D questions for mobility, self‐care, and usual activities. In an exploratory analysis, we examined change in function according to baseline frailty level as prior trials have shown difference in physical function according to frailty level at enrollment (Butt, Dewan, Merkely, et al., 2022). Finally, we stratified the main efficacy endpoint of the CANTOS trial (nonfatal myocardial infarction, nonfatal stroke, or cardiovascular death) according to baseline frailty status.

### 2.3 Frailty

Although there are multiple tools that can be used to assess frailty, in CANTOS, we primarily defined frailty according to the accumulation of deficits definition of frailty developed by Rockwood and Mitnitski (2007) (Mitnitski et al., 2001). This was done both for convenience of available data and to address the geroscience hypothesis that canakinumab affects multiple health‐related deficits that are associated with frailty. The Rockwood Frailty Index (FI) has been used in prospective and retrospective studies of diverse populations around the world (Ambagtsheer et al., 2018; Blodgett et al., 2015; Clegg et al., 2016; Orkaby, Lunetta, et al., 2018; Orkaby, Nussbaum, et al., 2018), and is a particularly useful tool to characterize older adult populations in clinical trials when a direct measure of frailty such as the Fried physical phenotype is not available (Butt, Dewan, Jhund, et al., 2022; Butt, Dewan, Merkely, et al., 2022; Orkaby et al., 2017; Pajewski et al., 2016; Warwick et al., 2015). The Rockwood theory of frailty posits that deficits in health accumulate over the lifespan. These deficits can be counted to generate a FI and determine an individual's frailty status.

To be included in the FI, variables must (1) be related to health status, (2) increase in prevalence with age, (3) not saturate in the population (e.g., presbyopia), and (4) include a range of systems such as cognition, function, and morbidity (Searle et al., 2008). For repeated measures of frailty, identical items should be assessed at each frailty assessment. A minimum of 30 deficits is typically evaluated. A total of 34 variables were included in the CANTOS FI, covering domains related to functional status, cognition, mood, comorbidities, laboratories, and weight (Ellis et al., 2020; Pajewski et al., 2016; Searle et al., 2008; Warwick et al., 2015) (Table S1). Because all participants in CANTOS had a prior myocardial infarction (MI), MI or coronary artery disease was not included in the FI as this variable would be saturated.

The number of deficits present for each individual was divided by the total possible number of deficits, with an overall score ranging 0–1 (e.g., 3 deficits/34 = 0.1). The 99th percentile for most populations 0.7 (Clegg et al., 2013; Searle et al., 2008). Scores >0.2 are frail, 0.1–0.2 are prefrail and <0.1 are nonfrail (Pajewski et al., 2016). Population studies suggest that community‐dwelling older adults accumulate deficits at an average rate of 3% per year (Mitnitski et al., 2005). Clinically meaningful annual changes in an FI score are thought to be 0.019 (small change) and 0.057 (large change) (Jang et al., 2020).

In a sensitivity analysis, we calculated an alternative definition of frailty based on the Fried physical phenotype of frailty. The Fried definition includes five interrelated variables: ≥5 lbs of unintentional weight loss in the last 2 years, self‐reported exhaustion, low‐energy expenditure according to kcal or energy, slow walking speed, and weak grip strength (Fried et al., 2001). The study of osteoporotic fractures (SOF) definition has validated a simplified version of the Fried to only three items: intentional or unintentional weight loss of >5% over the past year; inability to get up from a chair without using arms; and self‐reported reduced energy level (Ensrud et al., 2008). A modified SOF score was available for all CANTOS trial participants: >5% weight loss since baseline, difficulty with mobility, and difficulty with usual activities.

### 2.4 Functional impairment

Self‐reported items from the EQ‐5D‐3L instrument were used to identify impairment in mobility, self‐care, or usual activity. Each variable was assessed as “no problems,” “some problems,” or “unable.”

### 2.5 Major cardiovascular events

The primary endpoint of the trial was time to the composite endpoint of MI, stroke, or cardiovascular death. All events were adjudicated by an adjudication committee who were blinded to the trial‐group assignments (Ridker et al., 2017).

### 2.6 Other covariates

At baseline, information on age, sex, race, ethnicity, smoking status, body mass index (BMI), and blood pressure was measured.

### 2.7 Analytic plan

Descriptive statistics of the sample were computed overall and by frailty status.

The FI was first validated by assessing the association between FI level and risk of mortality over follow‐up using Cox regression (Searle et al., 2008). We then examined the cross‐sectional relationship of frailty to the inflammatory biomarkers measured in the trial: hsCRP and IL‐6.

For the primary outcome of incident frailty, we excluded those who were frail at study baseline and conducted an interval‐censored survival analysis for incident frailty using FI >0.2 as the cut point for frailty. Cumulative incidence curves for drug group versus placebo were run using logistic regression, adjusting for time point, drug group, a variable indicating qualifying MI event, and a variable indicating randomization status. These curves were repeated stratified by on‐treatment reduction of CRP and IL‐6 by more than the median at 3 months after study drug initiation, as this has previously been shown to identify greater benefit for cardiovascular endpoints in the trial (Ridker et al., 2018). Proc mixed models were then run to assess the change in mean FI score over time in the overall trial cohort. In sensitivity analyses, models were stratified by age (<60 and ≥60 years), sex, and on‐treatment hsCRP and IL‐6, using baseline values compared with the median. To conduct this analysis, we categorized the on‐treatment group by the presence or absence of a clinically meaningful response to study drug; that is, notable reduction in CRP and/or IL‐6. In CANTOS, all participants with a CRP below 2 and/or IL‐6 below the median at 3 months were considered “responders.” Inclusion criteria required that all patients had CRP >2 at baseline, so all patients with a CRP below 2 at 3 months were considered responders. For IL‐6, we separated out the on‐treatment group by those above and below median IL‐6 as had been done previously in CANTOS (Ridker et al., 2018). Analyses were repeated using the modified SOF definition of frailty.

For the secondary outcome of functional impairment, change in self‐reported mobility, self‐care, and usual activity was plotted over time and then stratified by frailty status as baseline frailty has been shown to be an effect modifier of function (Butt, Dewan, Merkely, et al., 2022).

For the analysis of the main trial outcome stratified by frailty, we reran the primary intention to treat time to event analysis, using pairwise comparisons of individual dose groups with the placebo group, as well as any drug versus placebo and stratified the results by frailty level (nonfrail, prefrail, and frail).

All models were run in SAS 9.4.

## 3 RESULTS

Among 10,061 participants in CANTOS, 9942 (99%) had sufficient data to calculate an FI at baseline (Figure S1). Overall, median age was 61 (IQR 54–68, range 28–92); 74% of participants were male, 12% were Asian, 3% were Black, 80% were White, and 16% were Hispanic or Latino. At baseline, 23.5% were current smokers and median BMI was 29.8 (IQR 26.6–33.8) kg/m^2^. The distribution of the FI was right skewed with a maximal value of 0.46 (Figure S2). The median FI score was 0.12, and 13.3% of participants were frail at baseline. Tables S2 and S3 affirm comparability of the groups by randomization in the trial for the entire cohort and amongst those who are nonfrail.

Table 1 shows the trial population by frailty status at baseline. Those who were frail at baseline were more likely to be older, female, and White. They were also more likely to be overweight and have chronic health conditions, including metabolic syndrome, and higher median levels of inflammatory biomarkers. There was no difference in frailty distribution according to randomization group at baseline.

**TABLE 1:** Baseline characteristics of 9942 CANTOS participants by frailty category at baseline.

	Not frail, *n* = 4052	Pre‐frail, *n* = 4540	Frail, *n* = 1350	Total, *n* = 9942
Age—median (IQI)	59 (52, 66)	62 (55, 69)	64 (58, 71)	61 (54, 68)
Sex—*n* (%)				
Male	3186 (81.7)	3377 (71.5)	832 (62.9)	7395 (74.4)
Female	712 (18.3)	1345 (28.5)	490 (37.1)	2547 (25.6)
Race—*n* (%)				
Asian	669 (17.2)	429 (9.1)	55 (4.2)	1153 (11.6)
Black or African American	68 (1.7)	172 (3.6)	76 (5.7)	316 (3.2)
Other	264 (6.8)	227 (4.8)	48 (3.6)	539 (5.4)
White	2897 (74.3)	3894 (82.5)	1143 (86.5)	7934 (79.8)
Ethnicity—*n* (%)				
Hispanic or Latino	658 (16.9)	748 (15.8)	150 (11.3)	1556 (15.7)
Not Hispanic or Latino	3163 (81.1)	3834 (81.2)	1083 (81.9)	8080 (81.3)
Unknown	77 (2.0)	140 (3.0)	89 (6.7)	306 (3.1)
Smoking status—*n* (%)				
Never	1069 (27.4)	1427 (30.2)	402 (30.4)	2898 (29.1)
Former	1793 (46.0)	2251 (47.7)	660 (49.9)	4704 (47.3)
Current	1036 (26.6)	1044 (22.1)	260 (19.7)	2340 (23.5)
BMI—median (IQR)	28.1 (25.1, 31.3)	30.6 (27.4, 34.5)	33.1 (29.7, 38.2)	29.8 (26.6, 33.8)
Systolic BP—median (IQR)	127 (118, 137)	131 (121, 142)	132 (121, 144)	130 (120, 140)
Diastolic BP—median (IQR)	78 (72, 83)	79 (72, 85)	77 (69, 84)	79 (72, 84)
Anemia—*n* (%)	27 (0.7)	194 (4.1)	179 (13.5)	400 (4.0)
Atrial fibrillation—*n* (%)	96 (2.5)	476 (10.1)	313 (23.7)	885 (8.9)
Cancer—*n* (%)	5 (0.1)	12 (0.3)	19 (1.4)	36 (0.4)
Diabetes—*n* (%)	629 (16.1)	2366 (50.1)	1026 (77.6)	4021 (40.4)
Heart failure—*n* (%)	305 (7.8)	1179 (25.0)	666 (50.4)	2150 (21.6)
Hypertension—*n* (%)	2379 (61.0)	4260 (90.2)	1282 (97.0)	7921 (79.7)
Stroke—*n* (%)	45 (1.2)	265 (5.6)	189 (14.3)	499 (5.0)
Peripheral artery disease—*n* (%)	100 (2.6)	435 (9.2)	339 (25.6)	874 (8.8)
Osteoarthritis—*n* (%)	128 (3.3)	729 (15.4)	497 (37.6)	1354 (13.6)
Gout—*n* (%)	77 (2.0)	415 (8.8)	264 (20.0)	756 (7.6)
Dementia—*n* (%)	4 (0.1)	17 (0.4)	18 (1.4)	39 (0.4)
Depression—*n* (%)	64 (1.6)	433 (9.2)	353 (26.7)	850 (8.5)
Asthma/COPD—*n* (%)	152 (3.9)	662 (14.0)	423 (32.0)	1237 (12.4)
Osteoporosis—*n* (%)	22 (0.6)	95 (2.0)	64 (4.8)	181 (1.8)
Hemoglobin—median (IQR)	145 (136, 153)	142 (132, 151)	137 (125, 148)	143 (132, 152)
Creatinine—median (IQR)	80.4 (70, 91)	82 (69.8, 97.2)	89 (72.5, 115)	82 (70, 96)
Albumin—median (IQR)	44 (43, 46)	44 (42, 46)	43 (41, 45)	44 (42, 46)
High sensitivity CRP—median (IQR)	3.75 (2.60, 5.95)	4.35 (2.90, 7.25)	5.6 (3.35, 9.55)	4.2 (2.80, 7.05)
IL‐6—median (IQR)	2.23 (1.56, 3.47)	2.63 (1.84, 4.09)	3.42 (2.31, 5.64)	2.58 (1.78, 4.09)
Drug group—*n* (%)				
Placebo	1304 (33.5)	1587 (33.6)	422 (31.9)	3313 (33.3)
50 mg	853 (21.9)	1029 (21.8)	262 (19.8)	2144 (21.6)
150 mg	898 (23.0)	1035 (21.9)	331 (25.0)	2264 (22.8)
300 mg	843 (21.6)	1071 (22.7)	307 (23.2)	2221 (22.3)
Frailty score—median (IQR)	0.07 (0.06, 0.09)	0.14 (0.13, 0.17)	0.24 (0.23, 0.27)	0.12 (0.09, 0.17)

*Note*: Non frail: FI <0.1, Pre‐frail: FI 0.1 to <0.2, Frail FI ≥0.2.

Those who were prefrail and frail had a higher risk of mortality over the duration of the trial (Table S3). Specifically, the HR for mortality rose from 1.50 (95% CI 1.32–1.71) for those who were not frail to 6.51 (95% CI 5.82–7.28) for those who were frail at the start of the trial.

### 3.1 Incident frailty

**FIGURE 1:**
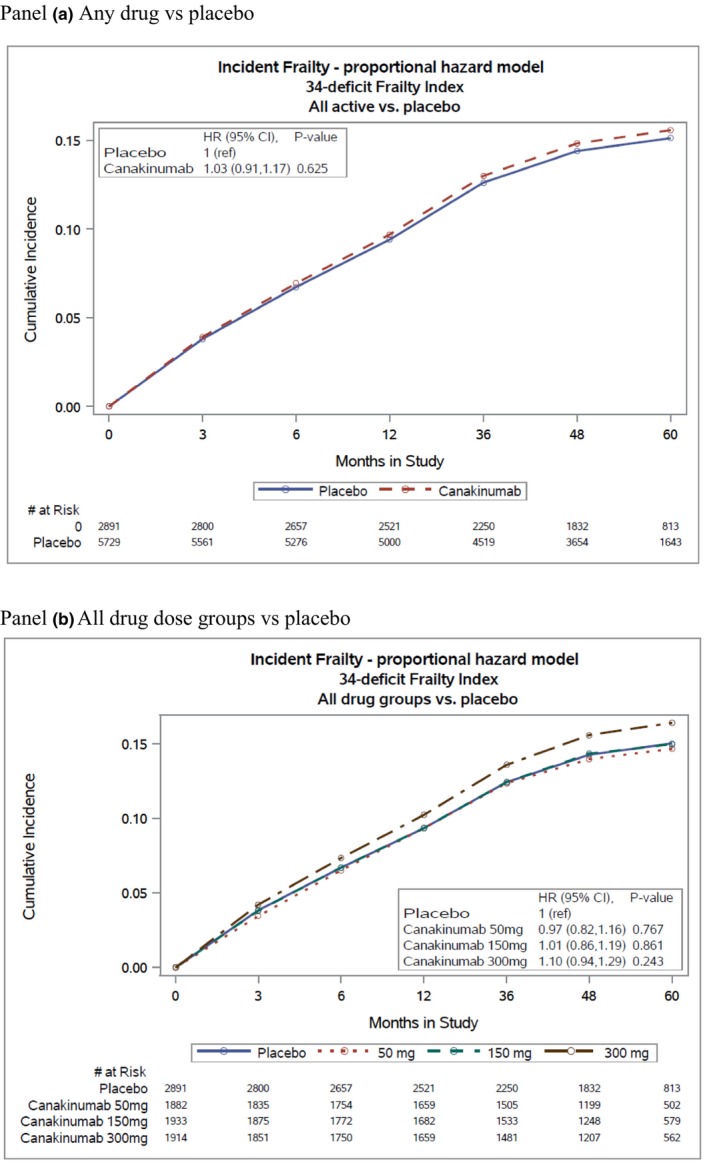
Effect of canakinumab on incident frailty (*n* = 8620).

Of the 8620 participants who were not frail at baseline, 1080 (12.5%) became frail during the 5‐year follow‐up period. There was no difference in rate of incident frailty according to randomization over time, comparing those allocated to any dose of canakinumab as compared to placebo or in comparisons of each dose separately to placebo (Figure 1, Panels A and B). As measured by the mSOF, at baseline, 1718 were frail (17.3%) and over follow‐up, there were 2208 new cases of frailty using the mSOF (Table S2). Using this alternative definition of frailty, we again observed no evidence that canakinumab reduced incidence frailty overall or at any dose compared with placebo (Figure S4). Results were unchanged in subgroup analyses by age, sex, and baseline inflammatory biomarkers (Table S5).

**FIGURE 2:**
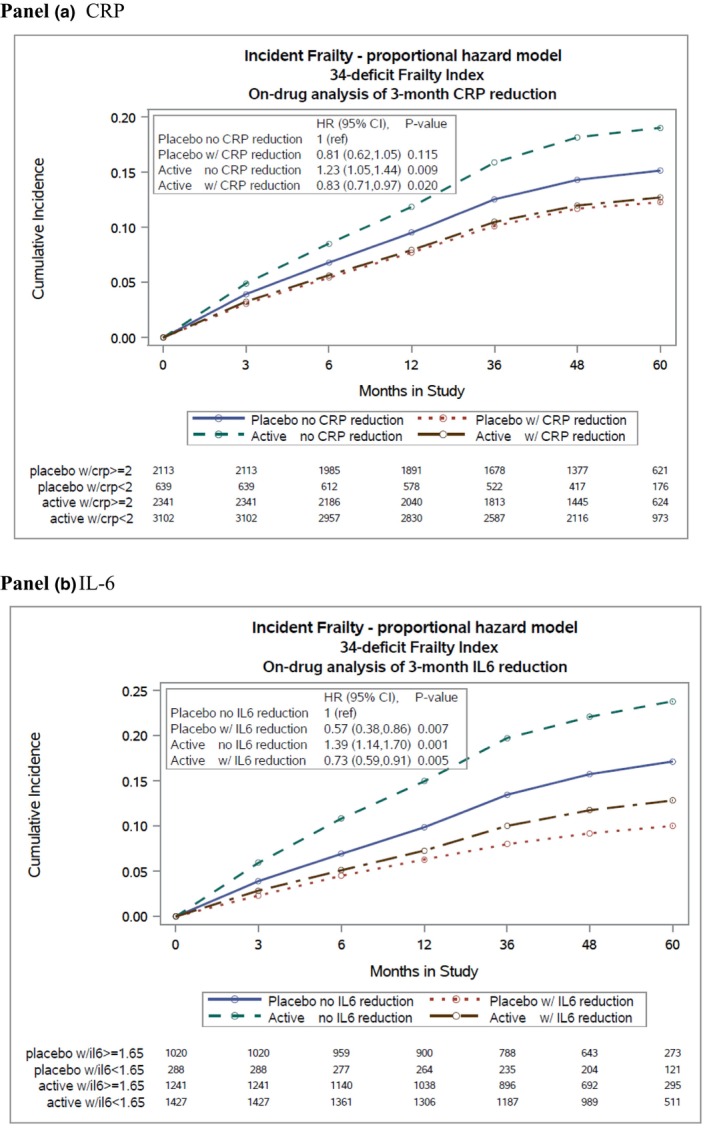
Effect of canakinumab on incident frailty according to on‐treatment CRP and IL‐6 reduction.

Cumulative incidence curves stratified by on‐treatment CRP and IL‐6 demonstrate a reduction in incident frailty among participants whose inflammatory markers were lower, irrespective of drug assignment category. For those assigned to placebo with CRP reduction, HR was 0.81 (95% CI 0.62–1.05) and for those assigned to active drug HR was 0.83 (95% CI 0.71–0.97) (Figure 2, panel A). Results were similar for IL‐6, as shown in Figure 2, panel B. For those assigned to placebo who experienced IL‐6 reduction, HR was 0.57 (95% CI 0.38–0.86), and for those assigned to active drug with IL‐6 reduction, HR was 0.73 (95% CI 0.59–0.91). Conversely for those assigned to canakinumab without CRP or IL‐6 reduction, there was an increased risk of incident frailty.

### 3.2 Change in frailty score

**FIGURE 3:**
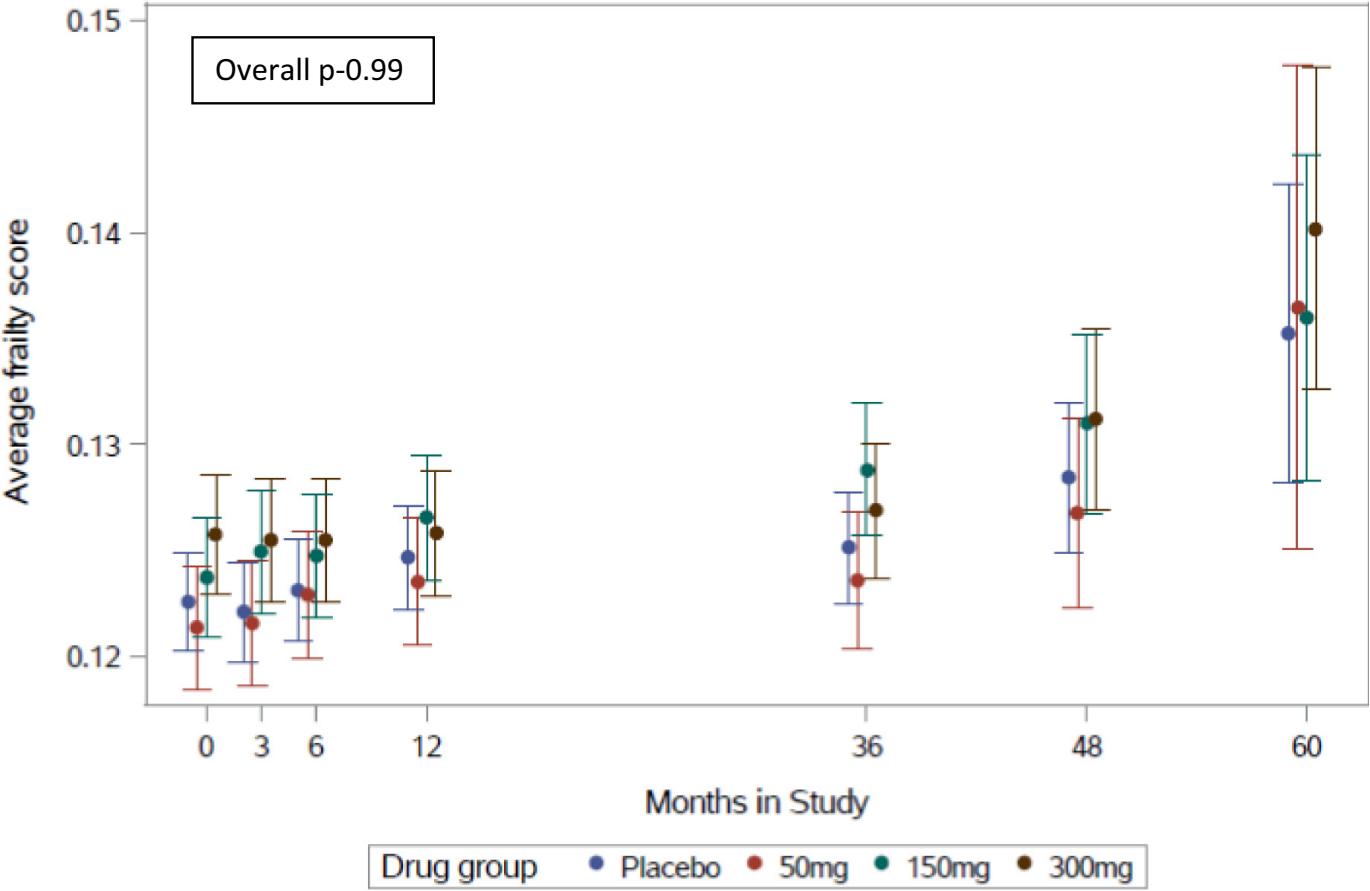
Effect of canakinumab on change in mean frailty score (*n* = 9942).

Mean frailty scores increased from 0.12 at baseline to 0.14 at the end of the trial. There was no difference in mean frailty score between randomization groups over time (Figure 3).

### 3.3 Physical function

Overall, there was no difference in self‐reported mobility, self‐care, or usual activities over the follow‐up period in those assigned to active drug or placebo (all *p* > 0.5) (Figure S4). Results were consistent when stratified by frailty level at baseline (not shown).

### 3.4 Major adverse cardiovascular events by baseline frailty

The primary trial outcome was rerun in the 9942 participants who had an FI available at baseline. Among those who were not frail 9.6% experienced a MACE outcome, while for those who were prefrail and frail, 15.8% and 27.4% experienced MACE, respectively. In the overall trial, the published HR (95% CI) compared with placebo for the 50‐, 150‐, and 300‐mg groups were 0.93 (0.80–1.07), 0.85 (0.74–0.98), and 0.86 (0.75–0.99); and for all doses versus placebo, it was 0.88 (0.79–0.97) (Ridker et al., 2017). After stratifying by frailty level at baseline, the same trends were evident among the frail at every level of frailty (Table 2). For the frail group, HRs for the 50‐, 150‐, and 300‐mg and all doses, each compared to placebo, were 0.91 (0.68–1.23), 0.86 (0.66–1.13), 0.84 (0.63–1.11), and 0.87 (0.71–1.08), respectively.

**TABLE 2:** Effect of canakinumab versus placebo on incident MACE, stratified by frailty.

Frailty level	Placebo (*N* = 3313)		50 mg (*N* = 2144)		150 mg (*N* = 2264)		300 mg (*N* = 2221)		All active drug (*N* = 6629)		*p*‐Trend^a^
	*N*	Rate^b^	*N*	Rate^b^	*N*	Rate^b^	*N*	Rate^b^	*N*	Rate^b^	
			HR^c^ (95% CI)		HR^c^ (95% CI)		HR^c^ (95% CI)		HR^c^ (95% CI)		
Not frail (<0.1), *n* = 4052	137	2.78	85	2.59	85	2.46	80	2.41	250	2.49	
	—		0.97 (0.74, 1.28)		0.88 (0.67, 1.15)		0.85 (0.64, 1.12)		0.89 (0.73, 1.10)		0.245
Prefrail (0.1 to <0.2), *n* = 4540	266	4.88	152	4.49	145	3.97	154	4.16	451	4.20	
	—		0.93 (0.76, 1.13)		0.81 (0.66, 0.99)		0.85 (0.70, 1.04)		0.86 (0.74, 1.00)		0.062
Frail (≥0.2), *n* = 1350	127	9.13	73	8.51	88	7.96	82	7.58	243	7.98	
	—		0.91 (0.68, 1.23)		0.86 (0.66, 1.13)		0.84 (0.63, 1.11)		0.87 (0.71, 1.08)		0.161

*Note*: MACE, major adverse cardiovascular events: nonfatal myocardial infarction, nonfatal stroke, or cardiovascular death.

^a^ Likelihood ratio test for trend from proportional hazard regression stratified by trial part.

^b^ Rates are per 100 person years.

^c^ Hazard ratio and confidence intervals from proportional hazard regressions stratified by trial part.

## 4 DISCUSSION

CANTOS has previously demonstrated that IL‐1b inhibition with canakinumab, compared with placebo, lowers the inflammatory biomarkers CRP and IL6 by 35 to 45 percent and significantly reduces rates of several clinical disorders associated with aging, including cardiovascular events, lung cancers, gout, anemia, and large joint osteoarthritis (Ferrucci & Fabbri, 2018; Jenny, 2012; Ridker et al., 2017; Ridker, MacFadyen, et al., 2017; Vallurupalli et al., 2020). In this post hoc analysis, however, we did not find that random allocation to canakinumab reduced incident frailty. Moreover, the relative efficacy of canakinumab for preventing cardiovascular events was not modified by baseline frailty status.

Although the underlying pathogenesis of frailty remains poorly defined, inflammation has been identified as a key contributor across diverse observational studies (Fulop et al., 2015; Liu et al., 2016; Reiner et al., 2009; Walston et al., 2002). A systematic review and meta‐analysis identified 32 cross‐sectional and three longitudinal studies (total *n* = 23,347) that examined the association between inflammation and frailty (Soysal et al., 2016). Both prefrailty and frailty were significantly associated with increased inflammatory biomarkers such as CRP, fibrinogen, IL‐6, and white blood cell count. However, in the three longitudinal studies, after adjustment for confounders, neither CRP nor IL‐6 were significantly associated with phenotypic frailty (meta‐analysis OR 1.06, 95% CI 0.78–1.44 and 1.19, 95% CI 0.87–1.62, respectively). In a study of aging mice who were treated with the angiotensin‐converting enzyme inhibitor enalapril or placebo, although enalapril treatment lowered inflammatory biomarkers, it did not significantly lower the overall risk of frailty measured according to a validated mouse FI (Keller et al., 2019). Taken together with the findings of the current study, the question of whether inflammation is a bystander in the development of frailty or is on the causal pathway and can therefore be intervened on remains an active area of investigation.

What are the implications of our study? It is possible that frailty may develop secondary to upstream hallmarks of aging phenomena that may contribute to inflammation, but which are not fully controlled by anti‐inflammatory interventions such as canakinumab. As such, future work should consider other hallmarks of aging, such as mitochondrial dysfunction, proteotoxic stress, senescence, and stem cell exhaustion that may be associated with frailty in those who also have inflammation, when considering therapeutics that target those mechanisms (Kulkarni et al., 2022; Lopez‐Otin et al., 2013; Sierra et al., 2021). Furthermore, this study cannot comment on the role of canakinumab for frailty prevention in those free of cardiovascular disease. The increased risk of frailty noted in those without reduction in inflammatory biomarkers raises a concern of side effects from the drug that may have worsened frailty status and should be considered in future studies.

While prior evidence from randomized treatment trials of anti‐inflammatory agents to reduce frailty is scant, observational studies have been inconsistent. In an observational study that examined the role of aspirin and NSAIDs on risk of frailty in 12,101 men, regular aspirin use over an average of 11 years was associated with a significant reduction in frailty (Orkaby et al., 2020, 2021), yet regular use of nonaspirin nonsteroidal anti‐inflammatory drugs (NSAID) was associated with an increased risk of frailty (Orkaby et al., 2022). Other observational studies have suggested that exercise and adherence to a healthy diet are associated with inflammatory biomarkers and frailty (Beavers et al., 2010; Bollwein et al., 2013; Clegg et al., 2013; Giugliano et al., 2006; Hoogendijk et al., 2019; Leon‐Munoz et al., 2014; Liu & Fielding, 2011; Ward et al., 2019). Each of these interventions influences the inflammatory process at different points and has other impacts on aging physiology, which may explain differential effects of each on frailty outcomes. Canakinumab blocks the inflammatory cascade upstream at pro‐inflammatory IL‐1β leading to downstream reduction in inflammatory proteins such as IL‐6, CRP. On the contrary, NSAIDs and Aspirin work downstream at the COX‐1 and COX‐2 enzyme sites. The additional impact of aspirin on platelet aggregation may explain some of the prior differences seen. On the contrary, regular exercise, for example, influences both inflammatory pathways as well as musculoskeletal and cardiopulmonary health and is a proven intervention for frailty (Clegg et al., 2013). It is possible that to impact clinical frailty, targeting more than a single physiologic pathway will be necessary.

We elected not to include CRP or IL‐6 in the frailty index for this study, as canakinumab is well‐known to lower these inflammatory biomarkers (Ridker et al., 2017, 2018), and our intent was to address whether other components of the FI were altered favorably by randomized drug allocation. We believe this decision was correct and provides an unbiased assessment of the ability of canakinumab to reduce frailty; indeed, when we included CRP in our frailty score on a post hoc basis, we observed a false‐positive finding due to score changes reflecting CRP reductions alone in the absence of any other frailty effects (data not shown). We thus caution against the use of frailty indices that include biomarkers of inflammation, at least in trials of targeted anti‐inflammatory agents.

It is notable that risk of a major adverse cardiovascular events was highest in those who entered the trial with a frailty index >0.2, consistent with the prior literature that has highlighted the increased risk of cardiovascular events among those who are frail (Damluji et al., 2021). That the main trial findings remained consistent when stratifying by frailty suggests that canakinumab should be considered as part of the treatment strategy even for those who are frail, and perhaps specifically in this high‐risk group, although with careful attention to the possibility of adverse events such as infections.

There are considerable strengths to our study, including its large sample size, prospective follow‐up, use of well‐validated frailty measures, and most important, random allocation to anti‐inflammatory therapy or placebo. Nonetheless, limitations of our study merit consideration. Although CANTOS participants had all survived myocardial infarction prior to trial initiation, participants were still healthier than the general older adult population. This is reflected in the maximal FI value in CANTOS of 0.46, which is lower than maximal FI values of 0.7 in US Medicare populations (Kim et al., 2017). Moreover, control of risk factors within CANTOS was far superior to that in the general population. This is particularly evident for hypertension where the mean systolic blood pressure is close to recommended guideline goals of 120 mmHg and for hyperlipidemia where the mean LDLC level was 82 mg/dL and over 90% were taking stain therapy. It is also possible that more time is needed for drug effects to influence frailty. For example, in the Look Ahead (Action for Health in Diabetes) study that tested an intensive lifestyle intervention in patients with diabetes, those randomized to the intervention had a significant slower accumulation of deficits over 8 years of follow‐up (Simpson et al., 2020). Additionally, while comparability of the trial arms was largely preserved in our analysis (see Table S2), the exclusion of individuals with prevalent frailty at baseline alters the participant allocation to dose groups initially achieved by randomization, and it remains possible that unmeasured confounding may have influenced the results described here. Finally, 75% of the CANTOS cohort were men; while we have no reason to believe that results should vary by biological sex, consideration of this possibility is beyond the scope of our analysis.

## 5 CONCLUSION

Among stable adult patients with atherosclerosis, random allocation to interleukin‐1b inhibition with canakinumab as compared to placebo did not lower risk of incident frailty over 5 years. More randomized data are needed to understand the role of targeted anti‐inflammatory medications for prevention of frailty and functional decline in older adults.

## AUTHOR CONTRIBUTIONS

ARO, RB, and PMR were involved in concept and design. PMR was involved in data curation and resources & supervision. AT, JM, and TGT were involved in analysis. All authors were involved in interpretation and writing—review & editing. ARO was involved in writing—original draft.

## FUNDING INFORMATION

Novartis funded the CANTOS trial and the statistical analyses conducted for this manuscript. This post‐hoc analysis was investigator initiated. The funders approved the manuscript but were not otherwise involved in the conduct of this study. Dr. Orkaby is funded by VA CSR&D CDA‐2 award IK2‐CX001800 and reports consulting fees from Anthos Therapeutics. Ms. MacFadyen reports grants from Novartis during the conduct of the study. Dr. Ridker served as the Principal Investigator of the CANTOS trial which was funded by Novartis. Dr. Ridker has received institutional research grant support from Novartis, Kowa, Amarin, Pfizer, Esperion, NovoNordisk, and the NHLBI; has served as a consultant to Novartis, Flame, Agepha, AstraZeneca, Janssen, Civi Biopharm, Glaxo Smith Kline, SOCAR, Novo Nordisk, Omeicos, Health Outlook, Montai Health, New Amsterdam, Boehringer‐Ingelheim, RTI; Zomagen, Cytokinetics, Horizon Therapeutics, and Cardio Therapeutics; has minority shareholder equity positions in Uppton, Bitteroot Bio, and Angiowave; and receives compensation for service on the Peter Munk Advisory Board (University of Toronto), the Leducq Foundation, Paris FR, and the Baim Institute (Boston, MA).

## CONFLICT OF INTEREST STATEMENT

None declared.

## Supporting information


Appendix S1

## Data Availability

Qualified external researchers may request access to patient‐level data and supporting clinical documents. These requests are reviewed and approved by an independent review panel on the basis of scientific merit. All data provided is anonymized to respect the privacy of patients who have participated in the trial in line with applicable laws and regulations.
