# Supplementary material for: Effect of canakinumab on frailty: A post hoc analysis of the CANTOS trial
Source: Aging Cell. 2023 Nov 5;23(1):e14029. doi: 10.1111/acel.14029 (PMC10776110; doi:10.1111/acel.14029)
Supplement: Supplementary file 1 — Appendix S1 [file ACEL-23-e14029-s001.docx]

Online Supplement for:

**Effect of Canakinumab on Frailty and Function: A post-hoc analysis of the CANTOS Trial**

Ariela R Orkaby, MD MPH, Aerin Thomson, BS, Jean MacFadyen, BA, Richard Besdine, MD, Daniel E. Forman, MD, Thomas G Travison, PhD, Paul M Ridker, MD, MPH

Table of Contents

**Supplemental Figures:**

**Supplemental Figure 1.** PRISMA flowchart

**Supplemental Figure 2.** Distribution of 34-item CANTOS Frailty Index

**Supplemental Figure 3**. Effect of canakinumab on incident frailty using the modified SOF score

**Supplemental Figure 4.** Effect of canakinumab vs placebo on self-reported EQ-5D functional questions: mobility, self-care, and usual activities for the overall trial cohort

**Supplemental tables:**

**Supplemental Table 1.** Components of the 34-item CANTOS Frailty Index

**Supplemental Table 2.** CANTOS Participant demographics at randomization by drug/placebo group (entire cohort)

**Supplemental Table 3.** CANTOS Participant demographics at randomization by drug/placebo group (non-frail)

**Supplemental Table 4.** Association between FI and mortality

**Supplemental Table 5.** Effect of canakinumab on incident frailty: Subgroup analyses using proportional hazards models

**Supplement Figure 1.** PRISMA flowchart

10,061 CANTOS participants

119 excluded due to insufficient data to calculate the frailty index

9,942 with sufficient data to calculate a frailty index at baseline and included in the trajectory analysis

1,322 frail at baseline

8,620 not frail at baseline

And included in the incidence analysis

**Supplemental Figure 2.** Distribution of 34-item CANTOS Frailty Index

**Supplemental Figure** **3**. Effect of canakinumab on incident frailty using the modified SOF score

**Panel A**. Any drug vs placebo

**Panel B**. Drug group vs placebo

**Supplemental Figure 4**. Effect of canakinumab vs placebo on self-reported EQ-5D functional questions: mobility, self-care, and usual activities for the overall trial cohort

**Panel A. Mobility**

**Panel B. Self-Care**

**Panel C. Usual Activities**

**Supplemental Table 1.** Components of the 34-item CANTOS Frailty Index

| **#** | **Variable** | **Definition** |
| --- | --- | --- |
| *Comorbidities* | | |
| 1 | Anemia | Yes = 1, No =0 |
| 2 | Atrial Fibrillation | Yes = 1, No =0 |
| 3 | Cancer | Yes = 1, No =0 |
| 4 | Diabetes | Yes = 1, No =0 |
| 5 | Heart Failure | Yes = 1, No =0 |
| 6 | Hypertension | Yes = 1, No =0 |
| 7 | Stroke (Ischemic, Hemorrhagic or Other) | Yes = 1, No =0 |
| 8 | COPD/asthma | Yes = 1, No =0 |
| *Function* | | |
| 9 | Peripheral Artery Disease | Yes = 1, No =0 |
| 10 | Osteoarthritis | Yes = 1, No =0 |
| 11 | Osteoporosis | Yes = 1, No =0 |
| 12 | Gout | Yes = 1, No =0 |
| 13 | Mobility (EQ-5D) | No problems = 0  Some problems = 0.5  Confined to bed = 1 |
| 14 | Self-Care (EQ-5D) | No problems = 0  Some problems = 0.5  Unable = 1 |
| 15 | Usual Activity (EQ-5D) | No problems = 0  Some problems = 0.5  Unable = 1 |
| *Sensory* |  |  |
| 16 | Macular Degeneration | Yes = 1, No =0 |
| 17 | Diabetic retinopathy | Yes = 1, No =0 |
| 18 | Diabetic neuropathy | Yes = 1, No =0 |
| 19 | Pain (EQ-5D) | No pain = 0  Moderate pain = 0.5  Extreme pain = 1 |
| *Cognition and Mental Health* | | |
| 20 | Anxiety/Depression (EQ-5D) | None = 0  Moderate = 0.5  Extreme = 1 |
| 21 | Depression diagnosis | Yes = 1, No =0 |
| 22 | Dementia/Cognitive impairment diagnosis | Yes = 1, No =0 |
| *Vitals and labs* | | |
| 23 | BMI (underweight, BMI < 18.5 kg/m^2^) | BMI < 18.5 kg/m^2^ = 1  Otherwise = 0 |
| 24 | BMI (overweight) | BMI ≥ 25 but <30 kg/m^2^ = 0.5  BMI ≥ 30 kg/m^2^ = 1  Otherwise = 0 |
| 25 | Systolic BP high (>=180mmHg) | Yes = 1, No =0 |
| 26 | Diastolic BP high (>=90mmHg) | Yes = 1, No =0 |
| 27 | Hemoglobin (>18 or <10.5 g/dL) | Yes = 1, No =0 |
| 28 | Blood Urea Nitrogen (<3 or >7.5 mmol/L) | Yes = 1, No =0 |
| 29 | Creatinine (>150 or <40 µmol/l) | Yes = 1, No =0 |
| 30 | Albumin <3.5g/L | Yes = 1, No =0 |
| 31 | Sodium (<135 or >150 mmol/L) | Yes = 1, No =0 |
| 32 | Glucose (<2.8 or >11mmol/L) | Yes = 1, No =0 |
| 33 | Alanine Aminotransferase (<10 or >50 U/L) | Yes = 1, No =0 |
| 34 | Alkaline Phosphatase (<35 or>129 U/L) | Yes = 1, No =0 |

**Supplemental Table 2.** CANTOS Participant demographics at randomization by drug/placebo group (entire cohort)

| **Variable** |  | **Canakinumab** | | | |  |
| --- | --- | --- | --- | --- | --- | --- |
|  | **Placebo**  **(n=3313)** | **50mg**  **(n=2144)** | **150mg**  **(n=2264)** | **300 mg**  **(n=2221)** | **All doses**  **(n=6692)** | **Total**  **(n=9942)** |
| **Age – median (IQR)** | 61 (54,68) | 61 (54,68) | 61 (54,68) | 61 (54,68) | 61 (54,68) | 61 (54,68) |
| **Sex – n (%)** | | | | | | |
| **Male** | 2458 (74.2) | 1618(75.5) | 1694 (74.8) | 1625 (73.2) | 4937 (74.5) | 7395(74.4) |
| **Female** | 855(25.8) | 526 (24.5) | 570 (25.2) | 596 (26.8) | 1692 (25.5) | 2547 (25.6) |
| **Race – n (%)** | | | | | | |
| **Asian** | 384 (11.6) | 231 (10.8) | 277 (12.2) | 261 (11.8) | 769 (11.6) | 1153(11.6) |
| **Black/African American** | 106 (3.2) | 61 (2.8) | 66 (2.9) | 83 (3.7) | 210 (3.2) | 316 (3.2) |
| **Other** | 196 (5.9) | 104 (4.9) | 131 (5.8) | 108 (4.9) | 343 (5.2) | 539 (5.4) |
| **White** | 2627 (79.3) | 1748 (81.5) | 1790 (79.1) | 1769 (79.6) | 5307 (80.1) | 7934 (79.8) |
| **Ethnicity – n (%)** | | | | | | |
| **Hispanic or Latino** | 520 (15.7) | 341 (15.9) | 356 (15.7) | 339 (15.3) | 1036 (15.6) | 1556 (15.7) |
| **Not Hispanic or Latino** | 2700 (81.5) | 1748 (81.5) | 1830 (80.8) | 1802 (81.1) | 5380 (81.2) | 8080 (81.3) |
| **Unknown** | 93 (2.8) | 55 (2.6) | 78 (3.4) | 80 (3.6) | 213 (3.2) | 306 (3.1) |
| **Smoking status – n (%)** | | | | | | |
| **Never** | 953 (28.8) | 595 (27.8) | 679 (30.0) | 671 (30.2) | 1945 (29.3) | 2898 (29.1) |
| **Former** | 1601 (48.3) | 1022 (47.7) | 1054 (46.6) | 1027 (46.2) | 3103 (46.8) | 4704 (47.3) |
| **Current** | 759 (22.9) | 527 (24.6) | 531 (23.5) | 523 (23.5) | 1581 (23.8) | 2340 (23.5) |
| **BMI, median (IQR)** | 29.7 (26.6-33.8) | 29.9 (26.6-33.9) | 29.8 (26.5-33.7) | 29.8 (26.5-33.8) | 29.9 (26.6-33.8) | 29.8 (26.6-33.8) |
| **SBP, median (IQR)** | 130 (120, 141) | 130 (120, 140) | 130 (119, 140) | 129 (120, 140) | 130 (120, 140) | 130 (120, 140) |
| **DBP, median (IQR)** | 79 (72-84) | 79 (72-84) | 79 (71-84) | 79 (72-84) | 79 (72-84) | 79 (72-84) |
| **Anemia - n (%)** | 127 (3.8) | 79 (3.7) | 90 (4.0) | 104 (4.7) | 273 (4.1) | 400 (4.0) |
| **Atrial Fibrillation- n (%)** | 302 (9.1) | 185 (8.6) | 204 (9.0) | 194 (8.7) | 583 (8.8) | 885 (8.9) |
| **Cancer- n (%)** | 17 (0.5) | 6 (0.3) | 5 (0.2) | 8 (0.4) | 19 (0.3) | 36 (0.4) |
| **Diabetes- n (%)** | 1337 (40.4) | 850 (39.6) | 957 (42.3) | 877 (39.5) | 2684 (40.5) | 4021 (40.4) |
| **Heart Failure- n (%)** | 714 (21.6) | 446 (20.8) | 475 (21.0) | 515 (23.2) | 1436 (21.7) | 2150 (21.6) |
| **Hypertension- n (%)** | 2622 (79.1) | 1729 (80.6) | 1798 (79.4) | 1772 (79.8) | 5299 (79.9) | 7921 (79.7) |
| **Stroke - n (%)** | 155 (4.6) | 113 (5.3) | 120 (5.3) | 111 (4.9) | 344 (5.2) | 499 (5.0) |
| **Peripheral Artery Disease- n (%)** | 307 (9.3) | 209 (9.7) | 173 (7.6) | 185 (8.3) | 567 (8.6) | 874 (8.8) |
| **Osteoarthritis- n (%)** | 432 (13.0) | 257 (12.0) | 327 (14.4) | 338 (15.2) | 922 (13.9) | 1354 (13.6) |
| **Gout- n (%)** | 247 (7.5) | 155 (7.2) | 175 (7.7) | 179 (8.1) | 509 (7.7) | 756 (7.6) |
| **Dementia/Cognitive impairment- n (%)** | 11 (0.3) | 11 (0.5) | 9 (0.4) | 8 (0.4) | 28 (0.4) | 39 (0.4) |
| **Depression - n (%)** | 278 (8.4) | 178 (8.3) | 201 (8.9) | 193 (8.7) | 572 (8.6) | 850 (8.5) |
| **COPD/asthma - n (%)** | 418 (12.6) | 260 (12.1) | 302 (13.3) | 257 (11.6) | 819 (12.4) | 1237 (12.4) |
| **Osteoporosis - n (%)** | 60 (1.8) | 40 (1.9) | 47 (2.1) | 34 (1.5) | 121 (1.8) | 181 (1.8) |
| **Hemoglobin, median (IQR)** | 143 (133,152) | 143 (134,151) | 143 (132,152) | 142 (132,152) | 143 (132,151) | 143 (132,152) |
| **Creatinine, median (IQR)** | 82 (70,96) | 82 (71,97) | 82 (71,95) | 82 (70,97) | 82 (71,97) | 82 (70,96) |
| **Albumin, median (IQR)** | 44 (42,46) | 44 (42,46) | 44 (42,46) | 44 (42,46) | 44 (42,46) | 44 (42,46) |
| **High Sensitivity CRP, median (IQR)** | 4.1 (2.8,6.9) | 4.3 (2.8,7.1) | 4.3 (2.9,7.1) | 4.2 (2.9,7.2) | 4.2 (2.8,7.1) | 4.2 (2.8,7.1) |
| **IL-6 median** | 2.6 (1.8,4.1) | 2.5 (1.8,4.2) | 2.6 (1.7,4.1) | 2.6 (1.8,4.1) | 2.6 (1.8,4.1) | 2.6 (1.8,4.1) |
| **Frailty Index, median (IQR)** | 0.11 (0.08,0.17) | 0.11 (0.08,0.17) | 0.11 (0.08,0.17) | 0.13 (0.08,0.17) | 0.11 (0.08,0.17) | 0.11 (0.08,0.17) |
| **Non-frail (0.1), n (%)** | 1304 (39.4) | 853 (39.8) | 898 (39.7) | 843 (38.0) | 2594 (39.1) | 3898 (39.2) |
| **Pre-frail (>0.1-0.2), n (%)** | 1587 (47.9) | 1029 (48.0) | 1035 (45.7) | 1071 (48.2) | 3135 (47.3) | 4722 (47.5) |
| **Frail (>0.2), n (%)** | 422 (12.7) | 262 (12.2) | 331 (14.6) | 307 (13.8) | 900 (13.6) | 1322 (13.3) |
| **SOF score, median (IQR)** | 0 (0.0) | 1 (0.0) | 0 (0.0) | 1 (0.0) | 2 (0.0) | 2 (0.0) |
| **Non-frail (0), n (%)** | 2022(61.0) | 1339(62.5) | 1373 (60.6) | 1344 (60.5) | 4056 (61.2) | 6078 (61.1) |
| **Pre-frail (1), n (%)** | 702 (21.2) | 460 (21.5) | 508 (22.4) | 474 (21.3) | 1442 (21.8) | 2144 (21.6) |
| **Frail (≥2), n (%)** | 589 (17.8) | 344 (16.0) | 383 (16.9) | 402 (18.1) | 1129 (17.0) | 1718 (17.3) |

**Supplemental Table 3.** CANTOS Participant demographics at randomization by drug/placebo group (non-frail)

| **Variable** |  | **Canakinumab** | | | |  |  |
| --- | --- | --- | --- | --- | --- | --- | --- |
|  | **Placebo**  **(n=2891)** | **50mg**  **(n=1882)** | **150mg**  **(n=1933)** | **300 mg**  **(n=1914)** | **All doses**  **(n=5729)** | **Total**  **(n=8620)** | **p-value (treatment vs placebo)** |
| **Age – median (IQR)** | 61 (54,68) | 61 (54,68) | 61 (54,67) | 61 (54,67) | 61 (54,68) | 61 (54,68) | 0.79 |
| **Sex – n (%)** | | | | | | | 0.52 |
| **Male** | 2189 (75.7) | 1456(77.4) | 1484(76.8) | 1434(74.9) | 4374(76.3) | 6563(76.1) |  |
| **Female** | 702 (24.3) | 426(22.6) | 449(23.2) | 480(25.1) | 1355(23.7) | 2057(23.9) |  |
| **Race – n (%)** | | | | | | | 0.78 |
| **Asian** | 369(12.8) | 224(11.9) | 257(13.3) | 248(13.0) | 729(12.7) | 1098(12.7) |  |
| **Black/African American** | 81( 2.8) | 47( 2.5) | 46( 2.4) | 66( 3.4) | 159( 2.8) | 240( 2.8) |  |
| **Other** | 175( 6.1) | 95( 5.0) | 117( 6.1) | 104( 5.4) | 316( 5.5) | 491( 5.7) |  |
| **White** | 2266(78.4) | 1516(80.6) | 1513(78.3) | 1496(78.2) | 4525(79.0) | 6791(78.8) |  |
| **Ethnicity – n (%)** | | | | | | | 0.88 |
| **Hispanic or Latino** | 477(16.5) | 305(16.2) | 316(16.3) | 308(16.1) | 929(16.2) | 1406(16.3) |  |
| **Not Hispanic or Latino** | 2344(81.1) | 1539(81.8) | 1562(80.8) | 1552(81.1) | 4653(81.2) | 6997(81.2) |  |
| **Unknown** | 70( 2.4) | 38( 2.0) | 55( 2.8) | 54( 2.8) | 147( 2.6) | 217( 2.5) |  |
| **Smoking status – n (%)** | | | | | | |  |
| **Never** | 822(28.4) | 516(27.4) | 576(29.8) | 582(30.4) | 1674(29.2) | 2496(29.0) | 0.19 |
| **Former** | 1395(48.3) | 887(47.1) | 894(46.2) | 868(45.4) | 2649(46.2) | 4044(46.9) |  |
| **Current** | 674(23.3) | 479(25.5) | 463(24.0) | 464(24.2) | 1406(24.5) | 2080(24.1) |  |
| **BMI, median (IQR)** | 29.3 (26.3-33.2) | 29.5 (26.4-33.4) | 29.3 (26.2-33.0) | 29.4 (26.2-33.2) | 29.4 (26.3-33.2) | 29.4 (26.3-33.2) | 0.79 |
| **SBP, median (IQR)** | 130 (120, 140) | 130 (120, 139) | 129 (119, 140) | 129 (119, 140) | 129 (120, 140) | 130 (120, 140) | 0.11 |
| **DBP, median (IQR)** | 79 (72-84) | 79 (72-84) | 79 (71-84) | 79 (72-84) | 79 (72-84) | 79 (72-84) | 0.94 |
| **Anemia - n (%)** | 72 (2.5) | 38 (2.0) | 52 (2.6) | 59 (3.1) | 149 (2.6) | 221 (2.6) | 0.75 |
| **Atrial Fibrillation- n (%)** | 197 (6.8) | 123 (6.5) | 124 (6.4) | 128 (6.7) | 375 (6.5) | 572 (6.6) | 0.64 |
| **Cancer- n (%)** | 10 (0.3) | 64 (0.2) | 0 | 3 (0.2) | 7 (0.1) | 17 (0.2) | 0.03 |
| **Diabetes- n (%)** | 1019 (35.2) | 645 (34.2) | 693 (35.9) | 638 (33.3) | 1976 (34.5) | 2995 (34.7) | 0.49 |
| **Heart Failure- n (%)** | 492 (17.0) | 320 (17.0) | 320 (16.5) | 352 (18.4) | 992 (17.3) | 1484 (17.2) | 0.73 |
| **Hypertension- n (%)** | 2214 (76.6) | 1475 (78.4) | 1476 (76.4) | 1474 (77.0) | 4425 (77.2) | 6639 (77.0) | 0.49 |
| **Stroke - n (%)** | 101 (3.5) | 69 (3.7) | 72 (3.7) | 68 (3.6) | 209 (3.6) | 310 (3.6) | 0.72 |
| **Peripheral Artery Disease- n (%)** | 202 (7.0) | 129 (6.9) | 95 (4.9) | 109 (5.7) | 333 (5.8) | 535 (6.2) | 0.03 |
| **Osteoarthritis- n (%)** | 286 (9.9) | 169 (9.0) | 189 (9.8) | 213 (11.1) | 571 (10.0) | 857 (9.9) | 0.91 |
| **Gout- n (%)** | 158 (5.4) | 100 (5.3) | 112 (5.8) | 122 (6.4) | 334 (5.8) | 492 (5.7) | 0.49 |
| **Dementia/Cognitive impairment- n (%)** | 8 (0.3) | 5 (0.3) | 6 (0.3) | 2 (0.1) | 13 (0.2) | 21 (0.2) | 0.66 |
| **Depression - n (%)** | 173 (6.0) | 98 (5.2) | 110 (5.7) | 116 (6.1) | 324 (5.7) | 497 (5.8) | 0.54 |
| **COPD/asthma - n (%)** | 283 (9.8) | 174 (9.2) | 193 (10.0) | 164 (8.5) | 531 (9.3) | 814 (9.4) | 0.44 |
| **Osteoporosis - n (%)** | 42 (1.5) | 29 (1.5) | 24 (1.2) | 22 (1.1) | 75 (1.3) | 117 (1.4) | 0.59 |
| **Hemoglobin, median (IQR)** | 143 (134,153) | 143 (135,151) | 143 (134,153) | 143 (134,153) | 143 (134,152) | 143 (132,153) | 0.59 |
| **Creatinine, median (IQR)** | 81 (70,95) | 82 (70,95) | 81 (71,93) | 82 (70,95) | 81 (71,95) | 81 (71,95) | 0.33 |
| **Albumin, median (IQR)** | 44 (43,46) | 44 (42,46) | 44 (43,46) | 44 (42,46) | 44 (42,46) | 44 (42,46) | 0.65 |
| **High Sensitivity CRP, median (IQR)** | 4.0 (2.7,6.6) | 4.1 (2.8,6.7) | 4.1 (2.8,6.7) | 4.1 (2.8,6.7) | 4.1 (2.8,6.7) | 4.1 (2.8,6.7) | 0.54 |
| **IL-6 median** | 2.5 (1.7,3.8) | 2.4 (1.7,3.8) | 2.4 (1.7,3.9) | 2.5 (1.7,3.8) | 2.5 (1.7,3.8) | 2.5 (1.7,3.8) | 0.43 |
| **Frailty Index, median (IQR)** | 0.10 (0.07,0.15) | 0.10 (0.07,0.13) | 0.10 (0.07,0.13) | 0.10 (0.07,0.15) | 0.10 (0.07,0.13) | 0.10 (0.07,0.15) | 0.48 |
| **Frailty categorty** |  |  |  |  |  |  | 0.88 |
| **Non-frail (0.1), n (%)** | 1304 (39.4) | 853 (39.8) | 898 (39.7) | 843 (38.0) | 2594 (39.1) | 3898 (39.2) |  |
| **Pre-frail (>0.1-0.2), n (%)** | 1587 (47.9) | 1029 (48.0) | 1035 (45.7) | 1071 (48.2) | 3135 (47.3) | 4722 (47.5) |  |

**Supplemental Table 4.** Association between FI and mortality

| **Frailty category** | **Total n** | **Events** | **HR (95% CI)** |
| --- | --- | --- | --- |
|  |  |  |  |
| robust (<.1) | 3898 | 225 | 1.50 (1.32-1.71) |
| pre-frail (>=.1 and <.2) | 4722 | 533 | 3.00 (2.75-3.26) |
| frail (>=.2) | 1322 | 309 | 6.51 (5.82-7.28) |

**Supplemental Table 5.** Effect of canakinumab on incident frailty: Subgroup analyses using proportional hazards models

| **Variable** | **At risk placebo** | **At risk active** | **Events placebo** | **Events active** | **HR** | **P-interaction** |
| --- | --- | --- | --- | --- | --- | --- |
| Frailty Index | | | | | | |
| Age <60 | 1292 | 2611 | 143 | 254 | 0.87 (0.71-1.07) | 0.044 |
| Age ≥60 | 1599 | 3118 | 212 | 471 | 1.41 (0.97-1.34) |  |
| Sex – male | 2189 | 4374 | 242 | 494 | 1.03 (0.88-1.20) | 0.878 |
| Sex – female | 702 | 1355 | 113 | 231 | 1.05 (0.84-1.31) |  |
| Baseline hsCRP below median | 1544 | 3006 | 164 | 488 | 1.15 (0.96-1.37) | 0.381 |
| Baseline hsCRP above median | 1347 | 2722 | 191 | 237 | 1.29 (1.06-1.56) |  |
| Baseline Il-6 below median | 743 | 1524 | 37 | 153 | 1.46 (1.02-2.10) | 0.806 |
| Baseline Il-6 above median | 660 | 1303 | 84 | 127 | 1.38 (1.05-1.82) |  |
|  |  |  |  |  |  |  |
| SOF frailty | | | | | | |
| Age <60 | 1198 | 2422 | 271 | 550 | 1.00 (0.87-1.16) | 0.864 |
| Age ≥60 | 1526 | 3078 | 458 | 947 | 1.02 (0.91-1.14) |  |
| Sex – male | 2080 | 4243 | 526 | 1024 | 0.94 (0.85-1.05) | 0.01 |
| Sex – female | 644 | 1257 | 203 | 473 | 1.22 (1.03-1.43) |  |
| Baseline hsCRP below median | 1460 | 2864 | 374 | 1046 | 1.05 (0.93-1.18) | 0.122 |
| Baseline hsCRP above median | 1264 | 2636 | 331 | 391 | 1.22 (1.05-1.41) |  |
| Baseline Il-6 below median | 706 | 1480 | 86 | 325 | 1.31 (1.03-1.66) | 0.335 |
| Baseline Il-6 above median | 651 | 1286 | 169 | 216 | 1.13 (0.92-1.38) |  |
